# Efficacy and Safety of TangWang Prescription for Type 2 Non-Proliferative Diabetic Retinopathy: A Study Protocol for a Randomized Controlled Trial

**DOI:** 10.3389/fphar.2021.594308

**Published:** 2021-03-15

**Authors:** De Jin, Yuehong Zhang, Yuqing Zhang, Wenjing Huang, Xiang Meng, Fan Yang, Qi Bao, Meizhen Zhang, Yanan Yang, Qing Ni, Fengmei Lian, Xiaolin Tong

**Affiliations:** ^1^ Department of Endocrinology, Guang’anmen Hospital, China Academy of Chinese Medical Sciences, Beijing, China; ^2^ Graduate School, Beijing University of Chinese Medicine, Beijing, China

**Keywords:** diabetic retinopathy, TangWang prescription, randomized controlled trial, traditional chinese medicine, protocol

## Abstract

**Background:** Diabetic retinopathy (DR) is one of the most common and severe microvascular complications of diabetes mellitus (DM), which results in blindness among adults worldwide. Presently, the efficacy of drug treatments for diabetic retinopathy (DR) is not satisfactory, thus urgently necessitating effective drug treatment measures. TangWang prescription (TWP) has been found to have retinal protection effects in previous clinical and basic research. However, there is a lack of rigorous, randomized, and controlled studies. This study aims to evaluate the efficacy and safety of TWP in delaying the development of DR.

**Methods:** This study is a randomized, double-blind, placebo-controlled, parallel-group, multicenter clinical trial, consisting of 384 participants to be randomized in a 1:1 ratio in the treatment and control groups. Furthermore, the treatment and control groups will be administered the TangWang prescription and the placebo, respectively, each at a dose of one bag twice a day. The study period will last for 48 weeks. The primary outcome measure will be the changes in the degree of retinal microvascular lesions before and after treatment. The secondary outcome will be changes in the degree of hemangioma, microvascular bleeding, microvascular leakage, macular edema, and vision. All statistical tests will be two-sided, and a *p* < 0.05 will be considered statistically significant.

**Discussion:** We hypothesize that the patients with DR will benefit from TangWang prescription, and in addition to the central random system and platform of dynamic information collection, the patients’ conditions will be monitored, and the data collected for analysis. If successful, this study will provide evidence that the TWP formulation delays in the progression of DR.

**Trial registration:** The design of this trial has been registered with the ClinicalTrials.gov (NCT03025399).

## Introduction

Diabetic retinopathy (DR) is one of the most common and severe microvascular complications of diabetes mellitus (DM). In 2020, according to global estimations, the prevalence of DM was 8.3% (International Diabetes Federation, 2019) while that in China was 11.6% in adults, and the number of patients was over 113 million, with the highest-ranking worldwide (Xu et al., 2013). Furthermore, DR shows an increasing trend yearly with DM (Wang et al., 2009; Varma et al., 2016). Several epidemiological studies have found that with a DM duration of 3, 5, 10, and 15 years, the incidence of DR was 8, 25, 60, and 80%, respectively (LeCaire et al., 2013; Azad et al., 2014; Chew et al., 2014; Raman et al., 2016). As for vision-threatening diabetic retinopathy, an estimate of the prevalence rate in global was 10.2% (28 million people) (Zhang et al., 2010). Eye diseases and the visual impairment due to DR have a wide range of effects, including the increase in the financial burden of patients, the decline in work capacity, and the sharp increase in the demand for social support (Coyne et al., 2004).

Glycemic control is the key modifiable risk factor associated with the development of diabetic retinopathy. The International Diabetes Control and Complications Study and United Kingdom Prospective Diabetes Study show that glycemic control effectively delays the occurrence and development of microvascular complications in type 2 DM (T2DM) (Intensive blood-glucose control UKPDS 33, 1998; Nathan et al., 1993). Moreover, blood pressure, blood lipid, pregnancy, inflammatory markers, and body mass index are important risk factors for the development of DR (Frank, 2004; Baker et al., 2016). On the top of control these risk factors, early detection, early diagnosis and early treatment are the key to preventing permanent retinal damage and even blindness caused by diabetic retinopathy. However, for nonproliferative diabetic retinopathy, depending on the severity of the diabetic retinopathy and the presence or absence of macular edema, the treatment decision is whether or not to opt for laser therapy. Panretinal photocoagulation (PRP) is not recommended for DR without macular edema, and the ETDRS study showed that early PRP was more likely to progress to moderate vision loss than delayed photocoagulation (Fong et al., 1999). Early PRP photocoagulation of NPDR showed adverse effects on vision and reduced visual field. For the early stage of DR (non-proliferative stage), the treatment options for these patients are very limited, and the clinical outcomes remain unsatisfactory (Stamper et al., 1978; Haritoglou et al., 2009).

In recent years, the medical management of diabetic retinopathy using Chinese medicine has been cause for concern. Pharmacological researches show that Chinese medicine exerts its neuroprotective functions by inhibiting cell apoptosis in diabetic retinopathy rats and antioxidizing reaction in the retinal tissue of diabetic rats (Liu et al., 2003; Zhang et al., 2018). TangWang prescription (TWP) is composed of five Chinese herbal medicines (Milkvetch root, Radix Salviae Miltiorrhizae, Cattail pollen, Cassia Twig, Pale Butterflybush flower). Previous studies have demonstrated that TWF formula can significantly protect the blood-retinal barrier (BRB), improve endothelial dysfunction and pathological morphology of the pancreas, as well as significantly reduce the positive expression of glucagon (Pang, 2018; Di, 2019). However, TWP directed therapy in combination with basic treatment has not yet been tested clinically. Therefore, we will perform a randomized, controlled trial to evaluate the therapeutic effect of TangWang prescription in delaying the disease progression of patients with NPDR.

## Materials and Methods

### Study Design

This study is designed as a randomized, double-blind, placebo-controlled multicenter clinical trial in accordance with the Helsinki Declaration. This trial is planned to test the hypothesis that TangWang prescription can delay in the progression of DR. A total of 384 participants will be randomly divided into two groups, which will both be administered basic treatments, consisting of oral calcium dobesilate capsules (Ebewe Pharma Ges.m.b.H.Nfg KG, 0.5 g three times per day). All the participants will be randomly assigned to either the treatment group or the control group. The study group will be received the intervention with oral calcium dobesilate capsules + TangWang prescription (one bag twice a day). And the control group will be received the intervention with oral calcium dobesilate capsules + the placebo (one bag twice a day) (Sichuan New Green Pharmaceutical Technology Development Co., Ltd.). Furthermore, diabetes education and diet, as well as rational control of blood glucose, lipids, and pressure will be performed in the two groups according to the American Diabetes Association guidelines.

**FIGURE 1:**
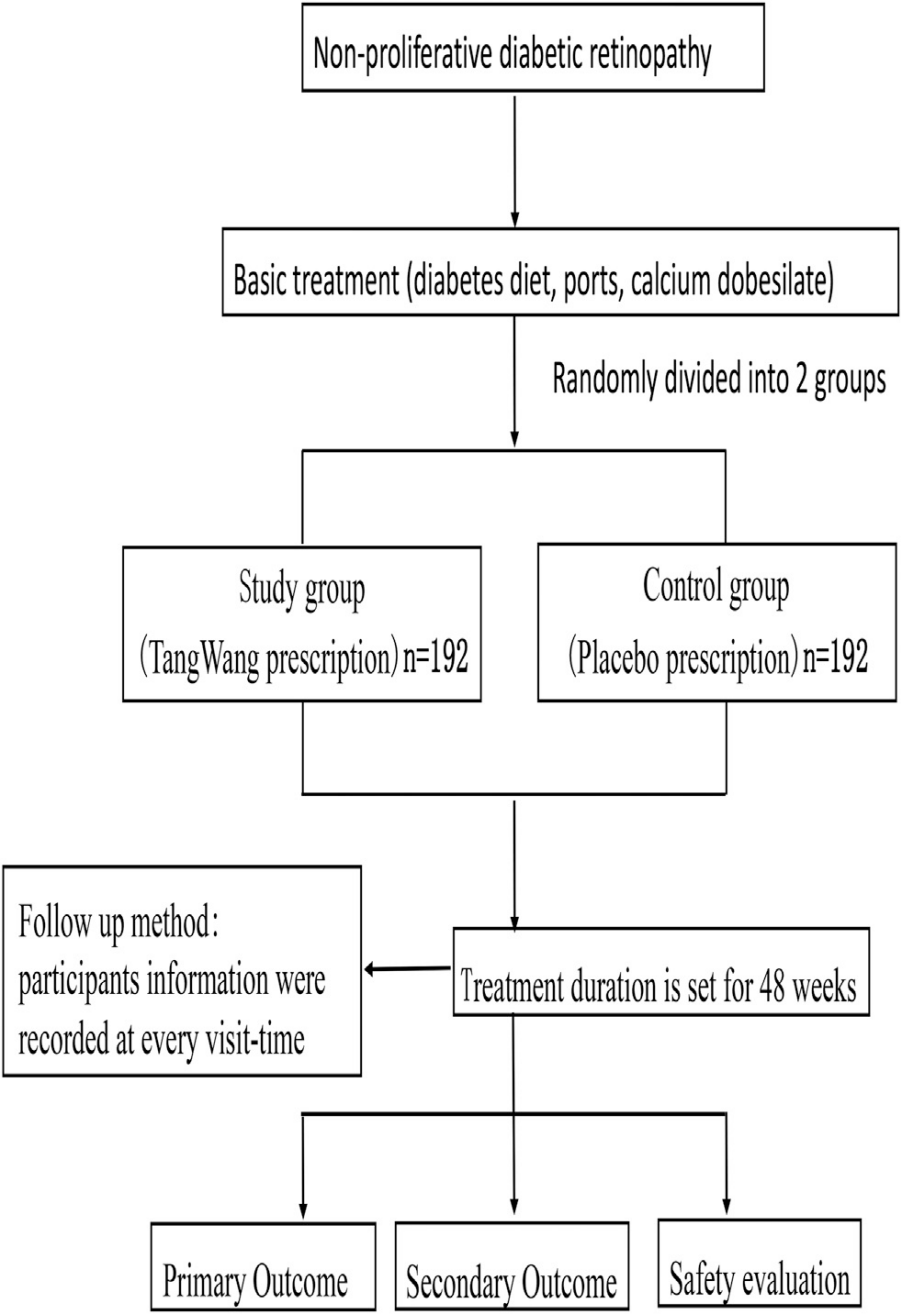
The trial schematic flow.

The central randomization system uses a randomly permuted block design, which includes subject screening, randomization, emergency exposure of blindness, designated drug, drug supply, and other functions. The treatment duration is set at 48 weeks, and the study medications will be labeled by pharmacists according to the randomized list prepared by the central randomization system. The trial schematic flow is illustrated in Figure 1.

### Participants

#### Inclusion Criteria

(1) Study participants must meet the diagnostic criteria of type 2 diabetic DR, and the required degree of disease is NPDR (Golozar et al., 2018).
  (1) Mild NPDR: microaneurysms only
  (2) Moderate NPDR: one or more of the following:
    i Hemorrhage (H)/microaneurysms moderate in 4-5 fields;
    ii or severer in one-field (or) definite intraretinal microvascular abnormality (IRMA) in 1-3 fields.
  (3) Severe NPDR: one or more of the following:
    - i More severe than moderate NPDR
    - ii Severe H/microaneurysms in 4-5 fields
    - iii IRMA > moderate in one-field
    - iv Definite venous beading (VB) in 2-3 fields.
(2) The participants must be between 30 and 70 years old.
(3) The participant must sign informed consent forms

Participants who have only one eye can meet the inclusion criteria, and the more severely damaged eye will be chosen when both eyes meet the inclusion criteria. Furthermore, the eye with a better clinical condition will be chosen when both eyes of the participant are at the same clinical stage.

#### Exclusion Criteria

(1) Patients with retinal photocoagulation, one or two eyes at the PDR phase, type I DM, other eye disease complications (such as glaucoma and cataracts, which could significantly interfere with the fundus examination, non-diabetic retinopathy, uveitis, amotio retinae, optic nerve diseases, and high myopia with fundus lesions).
(2) Combined severe primary diseases such as cardiovascular, liver, kidney, or hematopoietic system; serum transaminase levels more than double the normal value; serum creatinine greater than the upper limit of normal value; and patient with psychiatric conditions.
(3) Pregnancy, preparing for pregnancy or lactating.
(4) Patients are participating in other clinical research studies and the use of other drugs within a month of the trial.
(5) Patients who have taken other drugs to treat DR except for calcium hydroxide within a week of the study.
(6) Systolic blood pressure >160 mmHg or diastolic blood pressure >100 mmHg.
(7) Patients with diabetic ketosis, ketoacidosis, and severe infections within a month of the study.
(8) Patients who have abused alcohol, used psychoactive substances, abused drugs, or experienced drug dependence within 5 years of the study.
(9) According to the researcher’s judgment, patients with other diseases or conditions that might reduce study compliance or complicate their participation, such as frequent changes in the work environment, unstable living environment, and similar factors that could cause loss of contact.

#### Drop-Out Criteria

(1) Participants whose eyes progress to proliferative lesions or undergo laser photocoagulation.
(2) Participants whose vision decreases by three or more lines.
(3) Participants with certain complications or special physiological changes, which are not suitable for further study. Patients who experience some complications or physiological changes, rendering them unsuitable for further participation.
(4) Participants with poor study drug compliance of <80% of the requisite amount or >120% of the prescribed amount of medicine.
(5) Participants who break blinding or emergency unblinding of a participant’s information.
(6) Participants who use drugs prohibited by the study plan.
(7) Participants of reproductive age must receive contraceptive treatments. If the participants are pregnant, they would be considered dropouts.

### Voluntary Withdrawal of Participants

(1) Participants who do not express the desire to withdraw from the study but cease to accept medication and testing are considered withdrawn.
(2) Participants who cannot continue to participate in clinical research for personal reasons; perceive poor efficacy; exhibit intolerance due to adverse reactions; and for economic reasons.

Irrespective of the reason for withdrawal, the case record will be retained. The final test results will be used in the final analysis, and the curative effect and adverse reactions will be analyzed in the full dataset.

### Randomization and Data Collection

Patients who meet the criteria will be randomized into TWP and placebo groups. Randomized numbers will be generated by the central randomization system (http://www.tcmcec.net/crivrs/) established by the Clinical Evaluation Center of the Chinese Academy of Traditional Chinese Medicine. The randomized numbers will be ordered based on a given sequence, and patients will be divided into two groups according to the randomized numbers. The probability of participants being assigned to each group or center is not fixed but will be adjusted according to certain conditions. The electronic case report form (eCRF) was established by the Clinical Evaluation Center of the Chinese Academy of Traditional Chinese Medicine. All data will be recorded by the investigator in a CRF and eCRF. Furthermore, a unique random allocation is assigned using a system involving double blinding for participants and researchers. Every follow-up will be required to be logged into the central stochastic system by the investigator and into the central random system to obtain a drug number. The same platform will be used to collect information on the participants, random distribution, and assigned drugs to ensure random and blind methods are implemented and the test results are objective, to improve the quality of the clinical trial.

### Blinding Method

The central randomization system uses a randomly permuted block design, which includes subject screening, randomization, emergency exposure of blindness, designated drug, and drug supply. Blinding of the investigators, participants, statistician, staff in charge of data collection and clinical research associates will be blinded to the group assignments to avoid bias. In order to perform blinding, the placebo is processed into the same appearance, size, color, dosage form, weight, taste, and even smell like the same as the drug (TangWang prescription) in the treatment group. During the study, if participants experience severe adverse events, investigators will carry out an unblinding and perform urgent management accordingly. Statistician and staff in charge of data collection and clinical research associates, will all be third-party staff who did not involve in the design or patient recruitment of this study.

### Intervention Method

After grouping, participants will receive basic treatment including diabetes education, exercise and diet, as well as rational control of blood glucose, lipids, according to the 2017 diagnosis and treatment guidelines for the type 2 diabetes in China (Jia et al., 2019) and the 2020 American Diabetes Association guidelines (American Diabetes Association, 2020). In this work, according to the Chinese guidelines for exercise treatment of diabetes mellitus (Sun and Liu, 2013), If there are the following situations in patients: diabetic ketoacidosis, fasting blood glucose greater than 16.7 mmol/l, proliferative diabetic retinopathy, diabetic nephropathy and severe arrhythmia and transient ischemic attack, these patients could not be recommended to receive the exercise treatment. Furthermore, participants can choose the appropriate exercise style according to their preferences. All patients’ exercise patterns and exercise time will be recorded in detail. All the participants will be administered oral calcium dobesilate capsules (Ebewe Pharma Ges.m.b.H.Nfg KG, 0.5 g three times per day). The study group will be received TangWang prescription (one bag twice a day) and the control group will be treatment with placebo (one bag twice a day). The treatment duration is set at 48 weeks, and the study medications will be labeled by pharmacists according to the randomized list generated by the central randomization system. The management of TWP and the placebo will be assigned to a special pharmacologist in the hospital. The schedule of enrollment, interventions, and assessments is shown in Table 1.

**TABLE 1:** The schedule of enrollment, interventions, and assessments.

	Study period														
	Enrollment	Allocation	Post-allocation(w)												Close-out
Timepoint	-7-0		4	8	12	16	20	24	28	32	36	40	44	48	**t**_**x**_
Enrollment															
Eligibility screen	X														
Informed consent	X														
Allocation		X	X												
Interventions															
TangWang prescription															
Placebo															
Assessments															
Baseline	X	X	X												
Vital sign physical examination															
Routine ophthalmic examination fundus examination	X	X	X						X						X
Color fundus photography	X	X	X						X						X
Fluorescence fundus angiography	X	X	X						X						X
Optical coherence tomography	X	X	X						X						X
Vision															
Fasting blood-glucose, blood pressure															
Routine blood test and routine urine test stool routine examination	X	X	X						X						X
ECG, liver and kidney function, glycosylated hemoglobin, blood lipid	X	X	X			X			X			X			X

### Data Monitoring and Quality Control

The designated clinical research coordinator (CRC) will inspect and monitor the SOPs used in the research study. For the clinical research, a CRC will be appointed to regularly conduct on-site inspection visits to the hospitals to ensure strict adherence to all conditions of the research program and check the original data to ensure consistency with the CRF. This study will be supervised by a full-time Contract Research Organization (CRO) company. The study set up will include a data audit committee to audit the research data and research SOPs. Each audit will include at least one Project Manager and one project elite.

### Outcome Evaluation

#### Primary Outcome

The primary outcome of the study is the changes in the degree of retinal microaneurysm lesions before and after treatment. This will be determined according to the following scale: none, mild non-proliferative phase, moderate non-proliferative phase, severe non-proliferative phase, and proliferative phase, which are divided into aggravated, unchanged, and reduced conditions. The aggravated state is defined as retinal microaneurysm lesions with a degree of severity > grade 1 after treatment. The unchanged state was defined as a degree of retinal microaneurysm lesions before and after treatment that is unchanged, while the reduced state is defined as a degree of retinal microaneurysm lesions that is reduced by >1 grade after treatment.

### Secondary Outcome

1. Change in the number of microhemangioma in DR.
2. Change in the area of microaneurysm bleeding in DR.
3. Change in the area of microaneurysm leakage in DR.
4. Degree of change of macular edema in DR.
5. Change in vision before and after treatment.

Color fundus photography, fundus fluorescein angiography, and coherent optical tomography will be performed every 6x months. Furthermore, the Early Treatment Diabetic Retinopathy Study (ETDRS) international visual acuity test will be performed every month.

### Safety Evaluation

The blood pressure, fasting blood glucose, vital signs, physical examinations, vision tests, electrocardiogram, liver function (alanine aminotransferase, aspartate aminotransferase, gamma-glutamyl transferase, alkaline phosphatase, and total bilirubin) and renal function (blood urea nitrogen and urine creatinine) will be monitored monthly. Glycated hemoglobin and blood lipids will be determined every 3 months) while routine eye and fundus examinations, including intraocular pressure, anterior segment, lens, and vitreous body, optical coherence tomography every 6 months, vitreous body need to be monitored to describe the conditions of opacity. The schedule of enrollment, interventions, and assessments is shown in Table 2.

**TABLE 2:** The flow of the clinical data collection.

Visit	Project	Screening period/baseline	Visits 1–2,4–5,7–8,10–11	Visits 3,9	Visit 6	Visit 12
Visit time	-7-0 days	Medication 4, 8, 16, 20, 28, 32, 40, 44weeks ± 7 days	Medication 12, 36 weeks ± 7 days	Medication 24 weeks ± 7 days	Medication 48 weeks ± 7 days
Collect basic medical history					
Sign informed consent	×				
Fill in general information	×				
History of disease treatment	×				
Included inclusion and exclusion criteria	×				
vital Sign	×	×	×	×	×
physical Examination	×	×	×	×	×
merge Disease and medication	×	×	×	×	×
Diagnosis and monitoring					
Urine pregnancy test	×				
fasting Blood-glucose, blood pressure	×	×	×	×	×
glycosylated hemoglobin, blood lipids	×		×	×	×
Routine ophthalmic examination, fundus examination	×			×	×
Observation of effectiveness					
Color fundus photography	×			×	×
Fluorescence fundus angiography	×			×	×
Optical coherence tomography	×			×	×
Vision	×	×	×	×	×
Physico-chemical examination					
Routine blood test and routine urine test	×			×	×
Stool routine examination	×			×	×
Vital sign	×	×	×	×	×
Electrocardiogram, liver and kidney function	×		×	×	×
Adverse event		×	×	×	×
Other work					
Random grouping	×				
Distribute drug and patient’ diary card	×	×	×	×	
Recover drug, quantity statistics		×	×	×	×
Retrieve Patient’ diary card		×	×	×	×
Conclusion of research					×

Any adverse event (AE) will be dealt with carefully, analyzed, and measures will be taken to protect all participants. AEs will be recorded in the CRF according to the situation, and the record includes observations such as its duration, recurrence, and disappearance. If serious AEs (SAEs) occurs, the investigator must report them to higher authorities immediately and fill out the “Severe Adverse Event (SAE)” form. This study defines pregnancy as an SAE, indicating that:

(1)During the study, any pregnancy event will be recorded in the corresponding data form by the researcher according to the SAE reporting procedure within the shortest time when the pregnancy event is discovered, and follow-up the outcome.
(2)During the study, any abortion, whether accidental, natural or drug-induced will be reported as an SAE.
(3)Any congenital abnormalities or congenital disabilities of neonates of a study participant will also be reported as an SAE. When participants experience emergencies, the main investigator can break the blind condition according to the drug and symptoms, institute relevant measures, and record the information in the CRF carefully.

### Sample Size Calculation

According to preliminary research data (Lian et al., 2015), the rate of retinal microvascular disease was reduced by 3.6% in the placebo group, and we predicted that the rate of retinal microvascular disease would be reduced by 8.9% in the TangWang prescription group. Therefore, the sample content formula was estimated using two overall rate hypothesis tests: Assuming that α = 0.05 and β = 0.10 according to the one-sided test, to obtain the tabular value, uα = 1.64485 and uβ = 0.84162, the data will be placed in the formula to obtain an n = 166.82. Therefore, each group needs 167 participants and considering a drop-out rate of no more than 15%, and each group would need 192 participants, for a total of 384 participants.

Furthermore, the rate of retinal microaneurysms disease increased by 3.6% in the placebo group, and according to the above formula, we would need n = 108.78 patients. Therefore, each group requires 109 participants, and if we assume a drop-out rate of no more than 20%, each group needs 132 participants for a total of 264 participants. Based on these two conclusions, 384 participants will be included in this study.

### Statistical Analysis

All statistical tests will be two-sided tests, and a *p* < 0.05 will be considered statistically significant. Quantitative data to calculate the number of cases will be expressed as the mean, median, standard deviation, minimum, and maximum. Qualitative data will list the frequency and percentages. The demography, other baseline characteristics, and efficacy analyses will be analyzed using the full analysis set (FAS), while the per-protocol set (PPS) and the safety analysis will be performed using the safety analysis set (SS).

Quantitative data will be analyzed using the t-test/Wilcoxon rank-sum test, whereas the qualitative data will be analyzed using the X2 test/exact test. The main outcome measures will be analyzed using the X2 test for comparisons between groups, whereas secondary outcome and monitoring measures will be analyzed using the t-test/signed-rank test to compare the groups. The t-test/Wilcoxon rank-sum test will be used to compare the groups. All analyses will be performed using SAS version 9.3.

Three analysis sets will be used for the data analysis. The first is the FAS, which refers to an ideal set of subjects that are as close as possible to the principle of intentionality analysis (including all subjects randomized into the group and treated at least once). If the main variable is missing in any case, the results of the closest observation will be carried forward to substitute the absent test data. We will ensure that the number of participants in each group to be evaluated in the outcome at the end of the study is as consistent as possible before the start of the study. The second dataset is the PPS, consisting of all cases that are following the research plan. This set will consist of the data of participants with good compliance of 80–120% of the prescribed dose. The CRF contains the details of the case, and the main variables can be measured if the baseline variables are not missing, and there is no major violation of the test scheme. The third dataset is the SS, which will consist of the means of all participants who receive at least one treatment after randomization. The demographic, other baseline characteristics, and curative effect analyses, will be performed on the FAS and PPS, while safety will be analyzed using the SS.

## Discussion

The massive global increase in the incidence of diabetes illustrates the urgent need to inhibit diabetic retinopathy and other complications from progressing to advanced stages. According to our understanding, diabetic retinopathy progresses from non-proliferative diabetic retinopathy (NPDR) to more advanced forms of vision-threatening diabetic retinopathy that include proliferative diabetic retinopathy (PDR) and diabetic macular edema (DME) (Gerstenblith and Rabinowitz, 2012). Therefore, early detection and early treatment of NPDR are still the focus of diabetic retinopathy prevention and treatment. It is currently believed that the pathogenesis of DR is complex, and some aspects are still unclear. Studies have indicated the involvement of free radical damage, apoptosis of pericytes and retinal vascular endothelial cells, aggregation and adhesion of inflammatory factors, abnormal hemorheology, and lipid metabolism disorders, which occlude capillaries (Antonetti et al., 2012). Furthermore, the occluded capillaries are not perfused, which exacerbates local retinal ischemia and hypoxia, leading to a retinal vascular injury that promotes the occurrence and development of DR (Al-Kharashi, 2018). In addition, oxidative and hypertonic stress, as well as activation of inflammatory pathways triggered by late glycosylation end products and toll-like receptors, have been identified as key potential events (Wang and Lo, 2018). Chinese medicine believes that DR is a kind of “blood stasis” in the theory of Traditional Chinese Medicine, and medical management should be focused based on the core principle of clearing blood stasis. Blood circulation disorders in a high-sugar environment lead to blood stasis. The pathogenesis is mainly due to hemodynamic changes leading to microcirculation disorders, endothelial cell damage, platelet aggregation in the local area, which results in the smaller caliber of blood vessels, rough inner wall, abnormal microcirculation, higher viscosity of blood, red blood cell aggregation, slower blood flow, higher blood lipids, and accelerated formation of microthrombi, resulting in bruising (Lee et al., 2015). The blood changes from dynamic to static and manifests itself in circulatory disorders and damage to the affected tissues, as well as pathological changes such as inflammation, edema, exudation, hyperplasia, degeneration, sclerosis and necrosis of tissue cells (Wang et al., 2014; Lee et al., 2015).

TWP is a preparation that has “Qi boosting and stasis clearance” properties, which consisted of *Astragalus* membranaceus, Salvia miltiorrhiza, Pollen Typhae, Cassia Twig, Buddleja officinalis, which has exhibited a certain efficacy of clearing blood stasis. In the previous pharmacological studies, *Astragalus* membranaceus has been demonstrated that it ameliorated retinal neovascularization via the HIF-1α/VEGF signaling pathway, indicating it has the effect of protecting the retina (Thomas et al., 2017). Salvia miltiorrhiza had a certain protective effect on DR mice through the blood-ocular barrier (Zhao and Singh, 2018). For streptozotocin (STZ)-induced DR rats, Pollen Typhae can restore retinal ultrastructure and improve hemorheology indexes, as well as reduce abdominal capillary permeability (Popescu et al., 2018). Importantly, through animal experiments, we found that TWP not only play an anti-inflammatory role by inhibiting p38-MAPK and then inhibiting NF-κB pathway but also show the significantly reduce the positive expression of glucagon, which has a positive therapeutic advantage with the whole regulation, multi-target and multi-channel action (Pang, 2018; Di, 2019). Therefore, we believed that TWP could have a potential retinal protective effect on NPDR. Previously, our research team conducted a clinical trial and enrolled a total of 223 NPDR patients. Findings demonstrated the therapeutic value and safety of a danshen-containing Chinese herbal medicine in patients with diabetic retinopathy. Regarding its limitations including relatively short intervention cycle and lacks of evaluation index associated with macular edema, we design a randomized, double-blind, placebo-controlled multicenter clinical trial to evaluate further the potential clinical benefits of TWP towards diabetic retinopathy patients in NPDR.

We hypothesize that the patients with DR will benefit from TangWang prescription. If successful, this study will provide evidence that the TangWang prescription delays in the progression of DR. Although this protocol uses a rigorous randomized controlled trial to evaluate the effectiveness of the drug, there might also be potential limitations for this study. Firstly, in this multicenter trial, measurement errors in laboratory tests are also inevitable due to different medical equipment in several research centers. Secondly, there might be several individual differences in the subjects, possibly leading to different efficacy of the drug. Thirdly, the long treatment period and tight glucose control may lead to poor patient compliance, which might greatly influence the therapeutic effect. These questions should be explored more thoroughly in the future studies.

DR seriously threatens the vision of working-age people. For patients with NPDR, early treatment is of great significance. In recent years, the prevention and treatment of diabetic retinopathy by Chinese medicine has been a hot research topic. Up to date, this is the largest randomized controlled trial of Chinese medicine for diabetic retinopathy. In this protocol, a double-blind, randomized, placebo-controlled trial will be strictly implemented in multiple research centers. As the gold standard for evaluating drug efficacy, the implementation of the RCT will obtain high-level evidence-based medical evidence, which would fill up the gap in the treatment of early diabetic retinopathy.

## Abbreviations

AEs, adverse events; DM, diabetes mellitus; DR, diabetic retinopathy; NPDR, non-proliferative diabetic retinopathy; PDN, proliferative diabetic retinopathy; SAE, severe adverse event; T2DM, Type 2 diabetes mellitus; TCM, Traditional Chinese medicine.
